# Inhibition of 5α-Reductase, IL-6 Secretion, and Oxidation Process of *Equisetum debile* Roxb. ex Vaucher Extract as Functional Food and Nutraceuticals Ingredients

**DOI:** 10.3390/nu9101105

**Published:** 2017-10-10

**Authors:** Wantida Chaiyana, Chanun Punyoyai, Suvimol Somwongin, Pimporn Leelapornpisid, Kornkanok Ingkaninan, Neti Waranuch, Jukkarin Srivilai, Natthawut Thitipramote, Wudtichai Wisuitiprot, Roswitha Schuster, Helmut Viernstein, Monika Mueller

**Affiliations:** 1 Department of Pharmaceutical Science, Faculty of Pharmacy, Chiang Mai University, Chiang Mai 50200, Thailand; Chanew010@gmail.com (C.P.); suvimol_ampoo@hotmail.com (S.S.); pimleelaporn@gmail.com (P.L.); 2 Department of Pharmaceutical Chemistry and Pharmacognosy, Faculty of Pharmaceutical Sciences, Naresuan University, Phitsanulok 65000, Thailand; k_ingkaninan@yahoo.com (K.I.); netiw@nu.ac.th (N.W.); jukkarint@hotmail.com (J.S.); 3 School of Cosmetic Science, Mae Fah Luang University, Muang, Chiang Rai 57100, Thailand; nthitipramote@hotmail.com; 4 Department of Thai Traditional Medicine, Sirindhorn College of Public Health, Phitsanulok 65130, Thailand; wisuitiprot@hotmail.com; 5 Department of Pharmaceutical Technology and Biopharmaceutics, University of Vienna, Althanstrasse 14, Vienna 1090, Austria; roswitha.schuster@univie.ac.at (R.S.); helmut.viernstein@univie.ac.at (H.V.); monika.mueller@univie.ac.at (M.M.)

**Keywords:** *Equisetum debile*, chorioallantoic membrane assay, 5α-reductase, interleukin-6, lipid peroxidation

## Abstract

This study aims to investigate the biological activities related to hair loss of *Equisetum debile* extracts, including 5α-reductase inhibition, interleukin-6 (IL-6) secretion reduction, and anti-oxidation. *E. debile* extracts were obtained by maceration in various solvents. Crude extract (CE) was obtained by maceration in 95% ethanol. Chlorophyll-free extract (CF) was the CE which of the chlorophyll has been removed by electrocoagulation. Hexane extract (HE), ethyl acetate extract (EA), and ethanolic extract (ET) were fraction extracts obtained from maceration in hexane, ethyl acetate, and 95% ethanol, respectively. The extracts were investigated for inhibitory activity against 5α-reductase and IL-6 secretion. Total phenolic contents (TPC) were investigated and antioxidant activities were determined by means of 2,2′-azino-bis-3-ethylbenzothiazoline-6-sulfonic acid (ABTS), 2,2′-diphenyl-1-picrylhydrazyl (DPPH), and ferric reducing antioxidant power (FRAP) assays. The inhibition of lipid peroxidation was determined by the ferric thiocyanate method. The cytotoxicity of the extracts on dermal papilla cells and irritation test by hen's egg test chorioallantoic membrane assay were also investigated. All extracts could inhibit 5α-reductase and decrease IL-6 secretion in lipopolysaccharide-stimulated macrophage. The antioxidant activity of *E. debile* extracts was directly related to their TPC. ET which contained the highest TPC (68.8 ± 6.7 mg GA/g) showed the highest equivalent concentration (EC_1_) of 289.1 ± 26.4 mM FeSO_4_/g, TEAC of 156.6 ± 34.6 mM Trolox/g, and 20.0 ± 6.0% DPPH inhibition. However, EA exhibited the highest inhibition against lipid peroxidation (57.2 ± 0.4%). In addition, EA showed no cytotoxicity on dermal papilla cell line and no irritation on chorioallantoic membrane of hen’s eggs. In conclusion, EA was suggested as the most attractive ingredients for functional food and nutraceuticals because of the high inhibitory activity against 5α-reductase, IL-6 secretion, and lipid peroxidation inhibition.

## 1. Introduction

Androgenetic alopecia (common baldness) is recognized increasingly as a physically and psychologically harmful medical condition in which the pathogenesis is far from complete elucidation [1]. Generally, it is caused by aberrant hair follicle cycling and miniaturization of hair follicles, which depends on the presence of the androgenic hormones, including testosterone and dihydrotestosterone (DHT) [1,2,3]. In the human body, DHT is an enzymatic product converted from testosterone by the role of 5α-reductase. Since DHT is more active than testosterone, blocking the conversion of testosterone to DHT would reduce the androgenic effect. Thus, anti-androgenic drugs, which inhibit 5α-reductase or bind between DHT and androgen receptor, may be useful for protection from androgenetic alopecia [4].

The hair follicle is a cutaneous organ that remodels itself during cyclical periods of active hair growth (anagen), apoptosis-driven involution (catagen), hair shedding (exogen), and relative rest (telogen) [5]. Beside the androgenic hormones, the miniaturization of hair follicle might be explained by a shorter anagen cycle [6]. The hair follicle size and the duration of anagen phase indicate the length and the size of hair shaft, respectively [3]. The normal duration of anagen is around 2–6 years on average, and then it will turn to a short transitory period of catagen, in which the follicle will undergo apoptosis [7]. Therefore, one of the goals for treating androgenetic alopecia is to prolong the anagen [3].

Several cytokines are involved in the hair growth cycle, including interleukin-6 (IL-6). It has been reported that IL-6 is much more upregulated in balding dermal papilla cells comparing to non-balding dermal papilla cells [8]. Moreover, recombinant human IL-6 could inhibit the hair shaft elongation and suppressed proliferation of matrix cells in cultured human hair follicles, which lead to the premature onset of catagen during anagen phase [8]. Therefore, IL-6 might be a crucial inducer of hair loss in androgenetic alopecia.

Free radicals, which are highly reactive molecules with unpaired electrons that can directly damage various cellular components, might be another factor affecting the hair loss in androgenetic alopecia. Since the oxidation process leads to progressive damage of cellular structures, the ageing phenotype of hair manifests as a decrease in hair production [9]. It has been reported that lipid peroxides on hair follicles led to the early onset of the catagen in murine hair cycles [10]. Therefore, antioxidant compounds might be used to prolong the anagen phase and reduce the hair loss.

Nowadays, the potassium channel opener minoxidil and the dihydrotestosterone synthesis inhibitor finasteride have been used for the treatment of androgenetic alopecia [1]. However, these chemicals might cause some adverse reactions. Some patients using minoxidil encounter with fast or irregular heartbeat, rapid weight gain, bloating, flushing, redness of skin, swelling of feet or lower legs, etc., whereas, finasteride can cause cold sweats, confusion, dizziness, faintness, loss in sexual ability, etc. Therefore, there is an interest in finding new compounds for the treatment of androgenetic alopecia, especially from natural sources.

*Equisetum* Linn. is one of the most oldest living genera of vascular plant which comprises about twenty-five species [11]. The most investigated species was *Equisetum arvense* L. which has been widely used in traditional medicines for the treatment of hair loss [12]. The mixture of *E. arvense* shoot extract and mustard oil has been used as a hair tonic [13], whereas the mixture of *E. arvense* extract and other herbal extracts, such as bilberry, *Ginkgo biloba*, and saw palmetto, has been used as supplements for maintain a healthy hair follicle [14]. Additionally, the superior reduction of telogen effluvium duration in patients treated with herbal drug containing *E. arvense* extract (seven weeks) comparing to minoxidil solution (seven weeks) has also been reported [15].

Beside *E. arvense*, there are several species of *Equisetum* that have not been well studied. *Equisetum debile* Roxb. ex Vaucher (horsetail), a plant in the family Equisetaceae, is native to tropical South Asia [16]. It is widely distributed throughout the highland area of Thailand, especially 500 m above sea level. It has been used in folklore remedies by local highland people as diuretic, wound treatment, muscle relaxant, hair growth stimulant, and as anti-hair loss treatment. The decoction of *E. debile* has also been used for the hair strengthening [17]. Since *E. debile* and *E. arvense* are in the same family of Equisetaceae, they might have similar phytochemical compounds and biological activity. Some biological activities of *E. debile* extracts have been reported, such as antioxidant and antibacterial activity [18]. However, the phytochemical and anti-hair loss activities, such as the inhibitory activity against 5α-reductase and IL-6, have not yet been reported. Therefore, the aims of the present study were to investigate the anti-hair loss activities of fractionated *E. debile* extracts, including in vitro 5α-reductase inhibition, IL-6 secretion reduction, and antioxidant activity. Moreover, the irritation of the extracts on the chorioallantoic membrane of hen’s eggs was firstly reported in the present study.

## 2. Materials and Methods

### 2.1. Plant Materials

*E. debile* (horsetail) was collected from the highland area of Chiang Mai, Thailand, in January 2016. It was authenticated by Highland Research and Development Institute and its voucher specimen number 023221 was deposited in the Herbarium of the Faculty of Pharmacy, Chiang Mai University, Thailand. The aerial part of *E. debile* was washed and dried in the oven at a temperature of 40 °C. The dried plant material was ground into powder.

### 2.2. Chemical Materials

Nicotinamide adenine dinucleotide 2′-phosphate reduced tetrasodium salt (NADPH), Folin–Ciocalteu reagent, 2,2′-azino-bis-3-ethylbenzothiazoline-6-sulfonic acid (ABTS), 2,4,6 tripyridyl-s-triazine (TPTZ), 6-hydroxy-2,5,7,8-tetramethylchroman-2-carboxylic acid (Trolox), gallic acid, 2,2′-diphenyl-1-picrylhydrazyl (DPPH), lipopolysaccharide (LPS), linoleic acid, 4-(2-hydroxyethyl)piperazine-1-ethanesulfonic acid (HEPES) and 3-(4,5-dimethylthiazolyl-2) -2,5-diphenyl tetrazolium bromide (MTT), were purchased from Sigma-Aldrich (St. Louis, MO, USA). Potassium persulfate, potassium dihydrogen phosphate (KH_2_PO_4_), potassium chloride, sodium carbonate, sodium hydroxide, sodium acetate, sodium chloride, ferric chloride, ferrous sulfate, ferrous chloride, magnesium sulfate, and ammonium thiocyanate were purchased from Fisher Chemicals (Loughborough, UK). Tris base was purchased from Fisher Chem Alert (Fair Lawn, NJ, USA). RPMI-1640, Dulbecco modified eagle medium (DMEM), Penicillin/Streptomycin, L-glutamine, and trypan blue were purchased from Invitrogen™ (Grand Island, NY, USA). Hydrochloric acid and acetic acid were AR grade obtained from Merck (Darmstadt, Germany). Methanol, ethanol, hexane, ethyl acetate, dimethyl sulfoxide (DMSO), dichloromethane were AR grade and were purchased from Labscan (Dublin, Ireland). Sodium dodecyl sulfate (SDS) was purchased from EMD Millipore Corporation (Billerica, MA, USA).

### 2.3. Plant Extraction

#### 2.3.1. Crude Ethanolic Extraction 

The dried plant powder was macerated in 95% ethanol with some agitations for 72 h. The maceration was done triplicately, all the filtrates were pooled together and the solvent was removed under vacuum using rotary evaporator until dryness. The crude ethanolic extract (CE) was obtained and kept at 4 °C until further use.

#### 2.3.2. Chlorophyll-Free Extraction

CE was dissolved in 95% ethanol. Chlorophyll was then removed from the ethanolic solution by electrocoagulation using electrocoagulation reactor with aluminum probes for 3 h. The coagulated chlorophyll was removed by filtration through Whatman No. 1 filter paper. The solvent was then removed under vacuum using rotary evaporator until dryness. The chlorophyll-free extract (CF) was kept at 4 °C until further use.

#### 2.3.3. Fractionated Solvent Extraction

The dried plant powder was macerated in hexane with some agitations for 72 h. The maceration was done in triplicate and all filtrates were pooled together. The solvent was removed under vacuum using rotary evaporator until dryness and the fraction hexane extract (HE) was obtained. The plant residue was then fractionally macerated in ethyl acetate using the same method and the fraction ethyl acetate extract (EA) was obtained. Finally, the plant residue was fractionally macerated again in 95% ethanol using the above method and the fraction ethanolic extract (ET) was obtained. All dried extracts were kept at 4 °C until further use.

### 2.4. Determination of 5α-Reductase Inhibition

Anti-androgenic activity via the steroid 5α-reductase inhibition mechanism was evaluated using a label-free enzymatic inhibitory assay. The enzymatic activity was determined by analyzing the DHT formation after an enzymatic reaction using liquid chromatography–mass spectrometry (LC-MS) [19].

#### 2.4.1. Enzymatic Preparation

The enzyme homogenate from androgen-dependent prostate cancer cell, LNCaP cells (CRL-1740™ from American Type Culture Collection (ATCC), Manassas, VA, USA), was used as a source of 5α-reductase [20]. LNCaP cells expressing human steroid 5α-reductase were cultured in a 175 cm^2^ culture flasks at 37 °C under 5% CO_2_ humidified atmosphere. The medium was RPMI-1640 supplemented with 10% (*v/v*) fetal bovine serum and 100 U/mL penicillin G and 100 μg/mL streptomycin (Gibco, Paisley, Scotland). At ≥80% cell confluence, the medium was discarded, the cells washed with Tris-HCl buffer pH 7.4, and then scraped off, centrifuged at 1900× *g* for 10 min. Lysis buffer pH 7.4 was added to the cell pellet to give a cell suspension ≥9 × 10^7^ cells/mL. This was homogenised on ice using a sonication probe with 10 s pulse on, 10 s off for 1 min 40% amplitude (Sonics Vibracell™ VCX130 probe V18, Newtown, CT, USA). After that, glycerol (Invitrogen, Carlsbad, CA, USA) was added to be 20% (*v/v*) and the homogenate stored at −80 °C until use. In this step, glycerol is necessary for enzyme homogenate since it would act as a cryoprotectant to protect damaging protein structure from the ice crystal during the storage under −80 °C. The homogenate protein content was measured using Pierce bicinchoninic acid (BCA) protein assay (Pierce, Rockford, IL, USA), according to the manufacturer’s instructions and using bovine serum albumin (BSA, Sigma-Aldrich, St. Louis, MO, USA) as standard. The calibration curve of the BSA was plotted against the OD595 in the range of 10–500 μg/mL.

#### 2.4.2. Enzymatic Inhibitory Assay

The test samples were dissolved in DMSO and aliquots of these added to the assay solution. Assays were performed in 96-deep-well plates (Agilent Technologies, Santa Clara, CA, USA) covered by well-cap mats (Thermo Scientific, Waltham, MA, USA). The total volume of enzymatic reaction mixture was 200 µL, composed of test substance, 34.7 µM testosterone and 1 mM NADPH in Tris buffer pH 7.4. The reaction was started by adding 200 µL of homogenate enzyme (75 µg total protein) and incubated at 37 °C for 60 min. The reaction was stopped by adding 300 µL of hydroxylamine (10 mg/mL in 80% (*v/v*) ethanol) and incubating at 60 °C for 60 min for derivatization process. Then, the 96-well plate was centrifuged at 1700× *g* for 10 min using microplate centrifugation, and the supernatants transferred to another 96-well plate ready for injection into the LC-MS. Two control samples were used which were C1 and C2. Both controls contained the complete reaction mixture as described above but C1 was stopped before enzymatic incubation, whereas, C2 was stopped after 60 min of incubation. In the test sample, 10 µL of *E. debile* extract dissolved in DMSO was added instead of Tris-HCl buffer pH 7.4. However, in the blank, DMSO was used instead of Tris-HCl buffer pH 7.4. The DHT production was measured using LC­MS. The extracted ion chromatogram (EIC) of derivatized­DHT (*m/z* [M + H]^+^, 306.2428), the area under curve was used to calculate enzymatic inhibition:

%Steroid 5α-reductase inhibition = [1 − (Sample − C1)/(C2 − C1)] × 100
(1)

The standard steroid 5α-reductase inhibitor, finasteride (Sigma-Aldrich, St. Louis, MO, USA) was used as positive control (95 ± 2.2% inhibition at 1.5 µg/mL, triplicated).

#### 2.4.3. LC-MS Method for the Measurement of DHT

The Agilent 1260 Infinity Series HPLC system with an auto-sampler accommodating either two 108-vial trays or two 96-well plates (Agilent Technologies, Santa Clara, CA, USA) was used. The analytical reversed phase column was a Phenomenex Luna^®^ C18 (2) (150 mm × 4.6 mm, 5 µm) with a guard column (Phenomenex C18, 4 mm × 3 mm, 5 µm). The HPLC was connected with an Agilent 6540 UHD Accurate-Mass Q-TOF LC/MS (Agilent Technologies, Santa Clara, CA, USA), equipped with a dual electrospray ionization (ESI) in positive mode and *m/z* range 100–1200. Nitrogen was the nebulizing gas at 30 psi, and the drying gas (10 L/min; 350 °C). The mobile phase was 0.1% (*v/v*) formic acid in purified water (solvent A) and 0.1% (*v/v*) formic acid in acetonitrile (LC-MS grade, ACI Labscan, Bangkok, Thailand) as solvent B. The gradient program was used as follows; the initial mobile phase was 60% solvent B and 40% solvent A; solvent B was linearly increased up to 80% over 8 min then held constant for 4 min. Each run was followed by a 2 min post-run. The total run-time analysis was therefore 14 min with the column temperature controlled at 35 °C. The flow rate was 0.5 mL min^−1^ and the injection volume was 20 µL. Mass data were analyzed using Agilent Mass Hunter Qualitative Analysis software version B06.00.

### 2.5. Determination of IL-6 Secretion Inhibition in LPS-Stimulated Macrophages

#### 2.5.1. Macrophage Culture

Lipopolysaccharide (LPS) stimulated RAW 264.7 (mouse macrophage) cells were used to examine the effect of *E. debile* extracts on the inflammatory process. Dexamethasone, a well-known anti-inflammatory drug, was used as a positive control. The cell culture was performed following the method used in the previous study of Mueller et al. with slight modifications [21]. Briefly, RAW 264.7 cells were seeded at a density of 2 × 10^6^ cells per well in DMEM in 24 well plates, and incubated at 37 °C, 5% CO_2_ and 90% humidity for 24 h. On the following day, 1 µL of test compound in ethanolic solution were added, and further incubated under the same condition for 2 h. After that LPS was added to a final concentration of 1 μg/mL and further incubated in the same condition for 24 h. On the third day, the media was removed and centrifuged at 13,500× *g* for 10 min to remove cells. Supernatant was aliquoted and analyzed by ELISA. Cells which were not treated with LPS served as a negative control and cells incubated with ethanol and LPS served as a positive control, of which the secreted cytokines was defined as 100%. The IL-6 concentration in the cell supernatants (100 μL) was determined by ELISA according to the manufacturer’s protocol (R&D Systems, Minneapolis, MN, USA). All incubation steps were performed at room temperature. The optical density at 450 nm, corrected by the reference wavelength 570 nm, was measured with a Genios Pro microplate reader (Tecan, Crailsheim, Germany).

#### 2.5.2. Determination of the Cell Viability by MTT Assay

Simultaneous with the ELISA, the viability of LPS-stimulated cells was assessed by a MTT assay, based on the mitochondrial-dependent reduction of MTT to formazan. After removing the supernatant for ELISA analysis, MTT was added to the cells, and the cells were incubated for at 37 °C, 5% CO_2_ and 90% humidity for 2 h. The supernatant was then removed, and the cells were lysed with lysis buffer (10% (*w/v*) SDS in 0.01 N HCl). The optical density at 570 nm, corrected by the reference wavelength 690 nm, was measured using a Genios Pro microplate reader.

#### 2.5.3. Calculation of the IL-6 Secretion

The calculated concentrations of cytokines were normalized to MTT values to reduce any variation from differences in cell density. For a positive control, cells were treated with only LPS and the resulting amount of secreted cytokines was defined as 100%. The results from the experimental compounds were then calculated as a percent of this value. The entire inflammation assay, starting with cell seeding and LPS-induction, was performed in triplicate in three time independent experiment.

### 2.6. Determination of Total Phenolic Contents by Folin–Ciocalteu Method

Total phenolic contents of each extracts were determined by Folin–Ciocalteu method with some modifications [22]. Briefly, 20 μL of the sample solution in DMSO with the concentration of 1 mg/mL was mixed with 180 μL of 1:10 diluted Folin–Ciocalteu reagent and kept in room temperature for 4 min. Then 80 μL of saturated sodium carbonate solution (~0.7 M) was added and kept in room temperature for another 2 h. The absorbance was measured at 750 nm by using a multimode detector (Beckman Coulter DTX880, Fullerton, CA, USA). Gallic acid was used as a standard and the total phenolic contents were expressed as mg/g gallic acid equivalents (GAE). Total phenolic content was calculated using the following equation:

Total phenolic content (mg GA/g) = [(a − b) − 0.021]/0.0057
(2)

where a is an absorbance of sample solution with the present of Folin–Ciocalteu reagent and b is an absorbance of sample solution without the present of Folin–Ciocalteu reagent. The entire experiment was done in triplicate.

### 2.7. Determination of Antioxidant Activity

#### 2.7.1. 2,2′-azino-bis-3-ethylbenzothiazoline-6-sulfonic acid (ABTS) Assay

Each extract was tested for its ABTS radical cation (ABTS^•+^) scavenging activity by ABTS assay with some modifications [23]. Briefly, ABTS^•+^ was previously prepared by mixing 7 mM ABTS with 2.45 mM potassium persulfate (K_2_S_2_O_8_) and kept in the dark at room temperature for 16 h. On the experiment day, 20 μL of the sample solution in DMSO with the concentration of 1 mg/mL was mixed with 180 μL of 1:20 diluted ABTS^•+^ solution and kept in room temperature for 5 min. The absorbance was measured at 750 nm by using a multimode detector (Beckman Coulter DTX880, Fullerton, CA, USA). Trolox was used as a standard and the ABTS^•+^ scavenging activity was expressed as Trolox equivalent antioxidant capacity (TEAC) which was calculated using the following equation:

TEAC (mM Trolox/g) = [(a − b) − 0.7573]/ − 0.0145
(3)

where a is an absorbance of sample solution with the present of ABTS^•+^ solution and b is an absorbance of sample solution without the present of ABTS^•+^ solution. All experiments were done in triplicate.

#### 2.7.2. 2,2′-diphenyl-1-picrylhydrazyl (DPPH) Assay 

Each extract was tested for their radical scavenging activity against stable DPPH by DPPH assay with some modifications [24]. Briefly, 20 μL of the sample solution in DMSO with the concentration of 1 mg/mL was mixed with 180 μL of 167 μM DPPH solution and kept in the dark at room temperature for 30 min. The absorbance was measured at 520 nm by using a multimode detector (Beckman Coulter DTX880, Fullerton, CA, USA). The scavenging effect was calculated using the following equation:

% scavenging effect = {1 − [(a − b)/(c − d)]} × 100,
(4)

where a is an absorbance of 20 µL of ethanol and 180 µL of 167 µM DPPH mixture, b is an absorbance of 200 µL of ethanol, c is an absorbance of 20 µL of sample solution and 180 µL of 167 µM DPPH mixture, and d is an absorbance of 20 µL of sample solution and 180 µL of ethanol mixture. All experiments were done in triplicate.

#### 2.7.3. Ferric Reducing Antioxidant Power (FRAP) Assay

Each extract was tested for its reducing power by FRAP assay with some modifications [25]. Briefly, 20 μL of the sample solution in DMSO with the concentration of 1 mg/mL was mixed with 180 μL of freshly prepared FRAP solution, which contains 0.3 M acetate buffer (pH 3.6), 10 mM 2,4,6 tripyridyl-s-triazine (TPTZ) solution in 40 mM HCl, and 20 mM ferric chloride (10:1:1), and kept in room temperature for 5 min. The absorbance was measured at 595 nm by using a multimode detector (Beckman Coulter DTX880, Fullerton, CA, USA). Ferrous sulfate (FeSO_4_) was used as a standard and the ferric ions reducing power were expressed as equivalent capacity (EC_1_) which represented the amount of FeSO_4_ equivalents per mg of the sample. EC_1_ was calculated using the following equation:

EC_1_ (mg FeSO_4_/g) = [(a − b) − 0.0211]/0.0027 (5)

where a is an absorbance of sample solution with the present of FRAP solution and b is an absorbance of sample solution without the present of FRAP solution. All experiments were done in triplicate.

#### 2.7.4. Inhibition of the Lipid Peroxidation by the Ferric Thiocyanate Method

Each extract was tested for its inhibition against lipid peroxidation by thiocyanate method with some modifications [26]. Briefly, solutions containing 50 µL of the sample solution in DMSO with the concentration of 1 mg/mL, 50 µL of 50% linoleic acid in DMSO, 50 µL of 10% aqueous solution of ammonium thiocyanate (NH_4_SCN), and 50 µL of 2 mM ferrous chloride (FeCl_2_) solution, were incubated at 37 °C for 1 h. The absorbance was measured at 500 nm by using a multimode detector (Beckman Coulter DTX880, Fullerton, CA, USA). The inhibitory activity was calculated using the following equation:

% inhibition = {1 − [(a − b)/(c − d)]} × 100,
(6)

when a is an absorbance of linoleic acid, NH_4_SCN, and FeCl_2_ mixture, b is an absorbance of the solvents, c is an absorbance of sample solution, linoleic acid, NH_4_SCN, and FeCl_2_ mixture, and d is an absorbance of sample solution and the solvents. The entire experiment was done in triplicate.

### 2.8. Cytotoxicity of E. debile Extracts on Dermal Papilla Cells

#### 2.8.1. Dermal Papilla Cells Culture

Dermal papilla cells were purchased from Promo cell; Bio-med (Bangkok, Thailand). Frozen cells were thawed under water bath at 37 °C. The cells were suspended into follicle dermal papilla cell growth medium (PromoCell GmbH) to which was added fetal bovine serum (4% *v/v*), bovine pituitary extract (0.4% *v/v*), basic fibroblast growth factor (1 ng/mL) (PromoCell GmbH, Heidelberg, Germany). Cells were incubated under 37 °C, 5% CO_2_ with 95% of relative humidity. The cells were sub-cultured when they reached 80–90% confluence.

#### 2.8.2. Cytotoxicity and Cell Proliferation Testing

Ten thousands of dermal papilla cells per wells were incubated in 96 wells plate for 24 h under 37 °C and 5% of CO_2_. The cells were treated by the plant extracts dissolved in ethanol with various concentrations ranging from 1 to 500 µg/mL. After that, cells were re-incubated for another 24 h. MTT assay was used for determining cell viability. Fifty microliter of 1 mg/mL of MTT solution was added into each well and incubated for 3 h. Formazan crystal was produced by living cell and was then dissolved in DMSO. Absorbance was determined at 515 nm by using microplate reader. Cytotoxicity and cell proliferation were determined by comparing with controlled cells [27].

### 2.9. Irritation Test by Hen's Egg Test Chorioallantoic Membrane (HET-CAM) Assay

The irritation study was performed using hen’s egg test chorioallantoic membrane (HET-CAM) assay with slight modifications [28,29]. This experiment was one of the convenience and famous irritation studies since the ethical approval did not need to be applied when the age of animal’s embryo was less than half of the total incubation period. The hen eggs were obtained after fertilization from Faculty of Agriculture, Chiang Mai University. All eggs were incubated for 7 days in the hatching chamber with 37.5 ± 0.5 °C, humidity 55 ± 7%.

For preparation of the CAM, the air chamber of the egg was indicated by flooding the eggs with light. The egg shell was opened with an electric drill and the white egg membrane that appeared was removed. The samples dissolved in jojoba oil were exposed to the CAM, and the specific alterations of the membrane and its blood vessel network were examined as hemorrhage, lysis, and coagulation. The hemorrhage was observed as the bleeding out from blood vessels of the vascularized CAM. The lysis was indicated by a disappearance of small blood vessels on the CAM as a consequence either of bleeding, dystonia of these fine vessels, or real disintegration. The coagulation included either intravascular coagulation (thrombosis) or extravascular coagulation of proteins on the CAM, which normally increases the CAM opacity. The time of first occurrence of the three above-mentioned endpoints were registered within a maximum period of 5 min (300 s). From these data, an irritation index (IS) was calculated using the following equation:

*IS* = [(301 − *t(h)*)/300 × 5] + [(301 − *t(l)*)/300 × 7] + [(301 − *t(c)*)/300 × 9], (6)

where *t(h)* is the time (s) when the first vascular hemorrhage was detected, *t(l)* is the time (s) when first vascular lysis was detected, and *t(c)* is the time (s) when the first vascular coagulation was detected. The irritation score (*IS)* was then evaluated as follows: 0.0–0.9, no irritation; 1.0–4.9, mild irritation; 5.0–8.9, moderate irritation; and 9–21, severe irritation [30]. The blood vessel networks were observed again after 60 min to see the long term irritation. The pictures of the CAM were then captured under the microscope by Lumix digital camera (Panasonic, Beijing, China).

### 2.10. Statistical Analysis 

All data were presented as a mean ± standard deviation (S.D.). Individual differences were evaluated by *t* test or one-way ANOVA followed by post-hoc tests. In all cases, * denotes *p* < 0.05, ** denotes *p* < 0.01, and *** denotes *p* < 0.001 indicated statistical significance.

## 3. Results

### 3.1. E. debile Extracts

Five *E. debile* extracts, CE, CF, HE, EA, and ET, were obtained by maceration. All extracts showed almost the same external appearances, i.e. they were dark green, sticky, and semisolid. CE showed the highest yield (16.1%) and CF showed lower yield (7.3%) because of the chlorophyll remove. The fractionated extracts show small amount of the yield. HE and EA showed the comparable yields which were 3.6% and 4.2%, respectively. The smallest yield was obtained in ET (1.2%) since the nonpolar and semi-polar compounds had been previously extracted by hexane and ethyl acetate.

### 3.2. 5α-Reductase Inhibition

**Figure 1:**
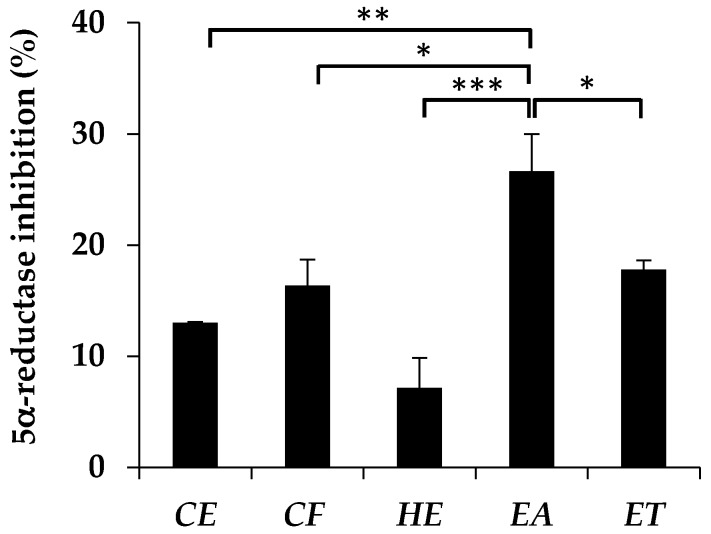
Inhibitory activity against 5α-reductase of crude extract (CE), chlorophyll-free extract (CF), fraction hexane extract (HE), fraction ethyl acetate extract (EA), and fraction ethanolic extract (ET) at the concentration of 0.1 mg/mL. Data are the mean value ± S.D. of three independent experiments (* denotes *p* < 0.05, ** denotes *p* < 0.01, and *** denotes *p* < 0.001).

The inhibitory activity against 5α-reductase of *E. debile* extracts are shown in Figure 1. EA possessed the significantly highest 5α-reductase inhibition among five extracts. Palmitic acid and phytosterols, which has been reported as a major component of the plant in the family of Equisetaceae [31,32], might be responsible for the 5α-reductase inhibitory activity of EA [33,34].

### 3.3. IL-6 Secretion Inhibition in LPS-Stimulated Macrophages

**Figure 2:**
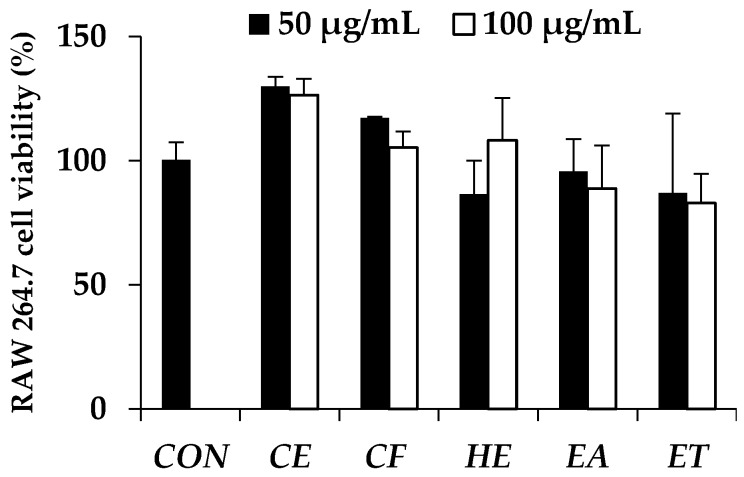
Cell viability of RAW 264.7 cell line after treatment with no extract (CON), crude extract (CE), chlorophyll-free extract (CF), fraction hexane extract (HE), fraction ethyl acetate extract (EA), and fraction ethanolic extract (ET) for 24 h. Data are the mean value ± S.D. of three independent experiments.

The IL-6 secretion levels were investigated in the RAW 264.7 cell line from which the cell viability after exposure to the *E. debile* extracts is shown in Figure 2. It was noted that the extracts had no toxicity on the cells because no significant difference was observed between the cell viability after exposure to each extracts for 24 h and the control cells which were not exposed to any extract (*p* > 0.05). Moreover, nearly 100% of cell viability was observed at the tested concentrations (50 and 100 µg/mL) of all extracts.

**Figure 3:**
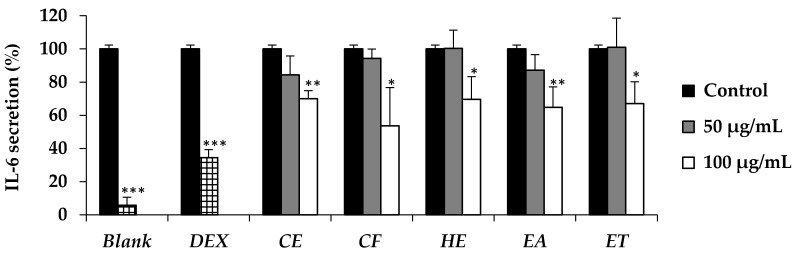
IL-6 secretion by the RAW 264.7 cell line without LPS treatment (Blank) and after being treated with LPS followed by 10 µM dexamethasone (DEX), crude extract (CE), chlorophyll-free extract (CF), fraction hexane extract (HE), fraction ethyl acetate extract (EA), and fraction ethanolic extract (ET) compared to control. Data are the mean value ± S.D. of three independent experiments. Asterisks denote values that were significantly different from the vehicle control (* denotes *p* < 0.05, ** denotes *p* < 0.01, and *** denotes *p* < 0.001).

The inhibitory activity against IL-6 secretion after treatment with various *E. debile* extracts are shown in Figure 3. There was no significant reduction of IL-6 secretion at low concentration (50 µg/mL) of all extracts but the obvious reductions were detected at high concentration (100 µg/mL) when compared to the cell control stimulated with LPS alone.

### 3.4. Total Phenolic Contents and Antioxidant Activity of E. debile Extracts

Total phenolic contents and antioxidant activity of each *E. debile* extracts is shown in Table 1. Individual antioxidants may act via multiple mechanisms. Therefore, no single assay would accurately reflect the antioxidant activity, especially in the plant extracts which contained several components. ET possessed the most potent antioxidant activity among five extracts which was obviously related to its highest total phenolic content, except for the inhibition of lipid peroxidation. The highest lipid peroxidation inhibition was possessed by EA. Besides, CF showed higher antioxidant activity comparing to CE in FRAP and DPPH assay because of the lower amount of chlorophyll. The likely explanation might be due to the production of potentially harmful singlet oxygen from chlorophyll. Chlorophyll was a major source of ROS production since the excitation energy can be transferred from photo-excited chlorophyll pigments to ^3^O_2_ that lead to the formation of singlet oxygen (^1^O_2_), superoxide (O_2_^•−^), and hydrogen peroxide (H_2_O_2_) [35].

**Figure 4:**
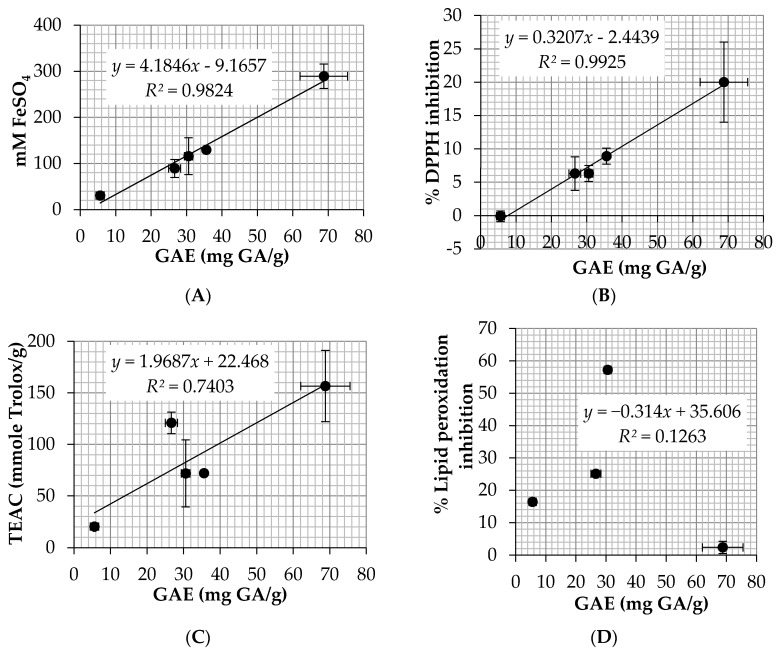
The correlations between total phenolic content and antioxidant activity from: (**A**) ferric reducing antioxidant power (FRAP) assay; (**B**) 2,2′-diphenyl-1-picrylhydrazyl (DPPH) assay; (**C**) 2,2′-azino-bis-3-ethylbenzothiazoline-6-sulfonic acid (ABTS) assay; and (**D**) ferric thiocyanate method. Data are the mean value ± S.D. of three independent experiments.

The correlations between total phenolic contents of five *E. debile* extracts and their antioxidant activities from various assays are shown in Figure 4. Linear positive relationships existed between the total phenolic contents and the antioxidant activity of the *E. debile* extracts from DPPH assay (*R*^2^ = 0.9925) and ABTS assay (*R*^2^ = 0.7403), which measured the radical scavenging activity. Similarly, a linear positive relationship was found in the FRAP assay (*R*^2^ = 0.9824), which measured the total reducing capacity of ferric ions. Although the mechanisms of FRAP assay was different from that of ABTS assay, the antioxidant results were comparative because the redox potential of Fe(III)-TPTZ was comparable with that of ABTS^•+^ [36]. Besides, the antioxidant results were in a good agreement with several previous studies which revealed that phenolic compounds had both abilities to scavenge free radicals and prevent generation of reactive oxygen species (ROS) by iron binding [37,38]. However, it was noted that the results from ABTS assay showed less correlation between the total phenolic contents and TEAC value. The likely explanation might be from the favor of water-soluble reactions in ABTS assay [36]. Therefore, the highest TEAC value of the fractionated extracts was found in ET, followed by EA and HE, which were finally extracted by ethanol (*ɛ* = 24.3), ethyl acetate (*ɛ* = 6.02), and hexane (*ɛ* = 1.9), respectively. Likewise, there was no correlation between total phenolic contents and antioxidant activities against lipid peroxidation (*R*^2^ = 0.1263) because the phenolic compounds, which were soluble well in water or polar solvents, were not well compatible with the lipid peroxidation test system.

### 3.5. Cytotoxicity of E. debile Extract on Dermal Papilla Cells

**Figure 5:**
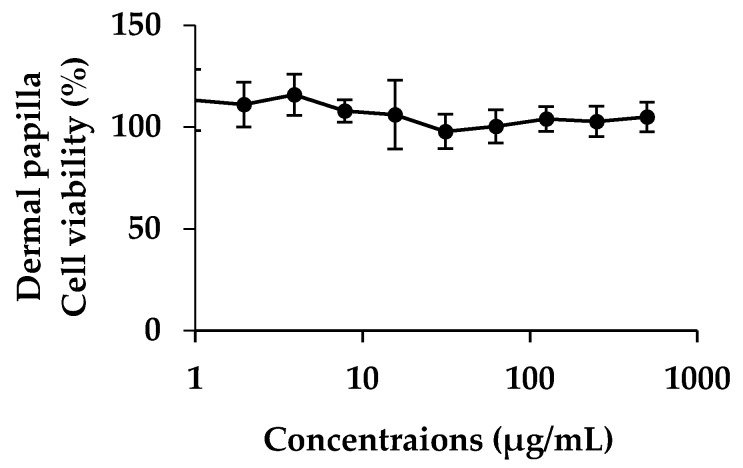
Dose–response curve of dermal papilla cell viability versus concentration of EA. Data are the mean value ± S.D. of three independent experiments.

According to the highest inhibitory activity against 5α-reductase and lipid peroxidation, as well as, high IL-6 secretion reduction, EA was the most attractive extract for anti-hair loss. Therefore, the cytotoxicity on dermal papilla cells of EA was investigated to confirm the safety of further uses. The human dermal papilla cell viability after exposure to EA for 24 h is shown in Figure 5. It is noted that EA was very safe since it had no toxic effect on human dermal papilla cells since nearly 100% of cell viability were observed even at high concentration.

### 3.6. Irritation Test by Hen's Egg Test Chorioallantoic Membrane (HET-CAM) Assay

**Figure 6:**
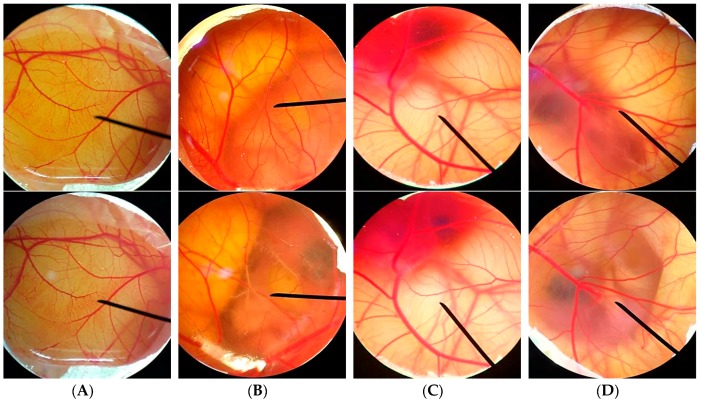
Photographs illustrating the effect of: (**A**) negative control (0.9% (*w/v*) NaCl); (**B**) positive control (1% (*w/v*) SLS); (**C**) vehicle (jojoba oil); and (**D**) EA (0.5% (*w/v*) solution in jojoba oil) in hen's egg test chorioallantoic membrane (HET-CAM) assay before applying the sample (upper) and at the end of the experiments of 60 min (lower)

The irritation results of HET-CAM assay are shown in Table 2. No irritation was observed in EA solution, which was the same result as observed in 0.9% (*w/v*) NaCl and the vehicle (jojoba oil). In contrast, moderate irritation was observed in 1% (*w/v*) SLS which have been known for the cause of scalp and skin irritation. The status of vessels before and after the experiments is shown in Figure 6. Only 1% (*w/v*) SLS could produce the lysis. It was noted that no hemorrhage, lysis, and coagulation were detected in the vessel exposed with EA solution after 60 min of exposure. Therefore, it might be concluded that EA was safe and would not cause the skin irritation. Since CAM is the vascularized respiratory membrane including veins, arteries, and capillaries, it has been used as a model for predicting the irritant effect of chemicals on the conjunctiva [39]. Consequently, EA would not cause the eye irritation and have a potential for the development of various anti-hair loss products, including hair tonic and hair spray.

## 4. Discussion

*E. debile* extracts have been extracted by maceration using various solvents. CE showed the highest yield since ethanol could extract a wide range of natural compounds from the plants, especially polar compounds. In the present study, electrocoagulation was used to remove chlorophyll from CE and yield CF. The yield was decreased from 16.1% of CE to 7.3% of CF. In addition, *E. debile* was fractionally extracted by using the non-polar solvent (hexane), semi-polar solvent (ethyl acetate), and polar solvent (95% ethanol). Therefore, the non-polar compound would be mostly found in HE, semi-polar compounds would be mostly in EA, and polar compounds would be mostly in ET. The yield of ET was very small when compared with that of CE since the nonpolar compounds and some semi-polar compounds have been removed.

The extracts were then investigated for anti-hair loss property. There are several biological mechanisms related to hair loss and the present study focused on 5α-reductase, IL-6, and oxidation process. 

The presence of DHT, which is converted from testosterone by the role of 5α-reductase, is related to aberrant of hair follicle cycling, miniaturization of hair follicles, and finally hair loss [1,2,3]. Therefore, the compounds that could inhibit 5α-reductase would be useful for anti-hair loss. The 5α-reductase inhibitory activity of *E. debile* extracts was firstly described in the present study. The results noted that, among the five *E. debile* extracts, EA possessed the significantly highest 5α-reductase inhibition. Although the activity was not as high as finasteride (95 ± 2.2% inhibition at 1.5 µg/mL), EA has a distinctive point as it was from natural source. The presence of palmitic acid as a major component of *E. debile* might be the explanation for the 5α-reductase inhibition [31]. The previous study has been reported that important configurations related to 5α-reductase inhibition included C12–C16 of the fatty acid chains [40]. Therefore, palmitic acid (C16:0) which was saturated C16 fatty acid exhibited the 5α-reductase inhibition [41]. In addition, the inhibitory activity could be more potent if there were the presence of a double bond in the molecule [40]. Additionally, there was a previous study reported that the fraction ethyl acetate extract of *E. debile* contained several phytosterols, such as stigmasterol and daucosterol [32], which could alter the metabolism of testosterone by inhibiting 5α-reductase. However, the evidence from animal studies suggested that a very high dose of phytosterol intake was needed to inhibit 5-alpha-reductase [34].

Beside the role of 5α-reductase and DHT, several cytokines are also related to the hair loss. IL-6 is one of the cytokines which has been more upregulated in balding dermal papilla cells [8]. In addition, IL-6 has been reported to inhibit the hair shaft elongation and suppressed proliferation of matrix human hair follicles cells and finally lead to the hair loss [8]. *E. debile* extracts (CE, CF, HE, EA, and ET) have been reported to reduce the IL-6 secretion in the present study. The active dose was detected at the concentration of 100 µg/mL. The results were in a good accordance with the previous study which reported that n-hexane and ethyl acetate extract of the aerial stems of *E. debile* composed of several phytosterols that could decrease aggregated LDL-induced secretion of IL-6 [32,42]. Since IL-6 has a broad effect on cells of the immune system and those not of the immune system and often displays hormone-like characteristics that affect homeostatic processes [43], *E. debile* extracts that could inhibit the IL-6 secretion might have several health benefits other than anti-hair loss.

Oxidation process is another pathway related to hair loss since free radicals could damage the hair follicle cellular structures and lead to a decrease in hair production [9]. There are several methods to investigate the antioxidant activity of natural compounds, including ABTS, DPPH, FRAP, and lipid peroxidation assay. However, the most relevant method related to hair loss was lipid peroxidation assay since it has been reported that lipid peroxides on hair follicles led to the early onset of the catagen which would lead to the hair loss [10]. 

Among 5 *E. debile* extracts, EA possessed the highest lipid peroxidation inhibition (57.2 ± 0.4%). EA also showed favorable antioxidant results in other assay with the EC_1_ of 115.7 ± 39.9 mM FeSO_4_/g and TEAC of 7.2 ± 3.3 mM Trolox/g. EA contained high level of total phenolic content (30.6 ± 1.2 mg GA/g) which was more abundant than CE (26.6 ± 1.7 mg GA/g). The explanation might be from the removal of non-polar compounds by the fractionated extraction method since HE which was the nonpolar extract contained very little amount of the phenolic content (5.6 ± 1.1 mg GA/g). The results were in a good agreement with the previous study which reported that a high and significant antioxidant activity was detected in the ethyl acetate fraction when comparing to an aqueous extract (infusion) and the major phenolic compounds responsible for the antioxidant activity were flavan-3-ol, kaempferol, and several phenolic acid derivatives [44]. Moreover, another study reported that quercetin, a flavonoid antioxidant, was isolated from ethyl acetate extract of the aerial stems of *E. debile* [32]. Therefore, quercetin would be one of the compounds that was responsible for the high antioxidant activity of *E. debile* extracts since it is able to scavenge highly reactive species such as peroxynitrite and the hydroxyl radical [45]. In conclusion, EA was the most attractive extract used for anti-hair loss since it showed the highest inhibitory activity against 5α-reductase, IL-6 secretion, and lipid peroxidation. Besides, the previous studies have been reported that fraction ethyl acetate extract of *E. debile* contained several phytosterols, flavonoids, and phenolic compounds which would be beneficial for health [32,46]. Moreover, EA was not toxic to the human dermal papilla cells and caused no irritation on HET-CAM. Therefore, EA might be used as functional food and nutraceuticals ingredients for anti-hair loss.

## 5. Conclusions

The present study demonstrated the inhibitory activity against 5α-reductase, IL-6 secretion, and oxidation process of *E. debile* extract. EA exerted the highest inhibition on 5α-reductase (26.6 ± 3.3%) and lipid peroxidation (57.2 ± 0.4%), whereas ET possessed the highest antioxidant activity (EC_1_ = 289.1 ± 26.4 mM FeSO_4_/g, TEAC = 156.6 ± 34.6 mM Trolox/g, and DPPH inhibition = 20.0 ± 6.0%), which was related to its highest amount of phenolic content (68.8 ± 6.7 mg GA/g). The results noted that EA was very safe since it showed no cytotoxicity on dermal papilla cell line and no irritation on chorioallantoic membrane of hen’s eggs. Therefore, EA might be an attractive ingredient for functional food and nutraceuticals for anti-hair loss because of the highest inhibitory activity against 5α-reductase, IL-6 secretion, and lipid peroxidation inhibition. However, the pharmacokinetic study of *E. debile* extract would be suggested for the further study.

## Figures and Tables

**Table 1:** Total phenolic contents and antioxidant activity of *E. debile* extracts.

Extracts	GAE (mg GA/g)	EC_1_ (mM FeSO_4_/g)	TEAC (mM Trolox/g)	%Inhibition	
				DPPH	Lipid Peroxidation
*CE*	26.6 ± 1.7 ^a^	89.2 ± 19.5 ^a^	12.1 ± 1.0 ^a^	6.3 ± 2.5 ^a^	25.1 ± 0.6 ^a^
*CF*	35.6 ± 0.5 ^b^	129. ± 2.0 ^b^	7.2 ± 0.3 ^a,b^	8.9 ± 1.2 ^a^	12.6 ± 0.9 ^b^
*HE*	5.6 ± 1.1 ^c^	30.3 ± 3.1 ^a^	2.0 ± 0.0 ^b^	ND	16.4 ± 1.1 ^c^
*EA*	30.6 ± 1.2 ^a,b^	115.7 ± 39.9 ^b^	7.2 ± 3.3 ^a,b^	6.3 ± 1.2 ^a^	57.2 ± 0.4 ^d^
*ET*	68.8 ± 6.7 ^d^	289.1 ± 26.4 ^c^	15.7 ± 3.5 ^a^	20.0 ± 6.0 ^b^	2.4 ± 1.9 ^e^

Results expressed as mean ± SD of triplicates. ND: not detected. CE: crude extract, CF: chlorophyll-free extract HE: fraction hexane extract, EA: fraction ethyl acetate extract, ET: fraction ethanolic extract. Superscript letters (^a^, ^b^, ^c^, ^d^, and ^e^) within the same column indicate significant (*p* < 0.05) differences of means between the groups based on Tukey’s HSD one-way ANOVA.

**Table 2:** IS score and irritation level form hen’s egg test chorioallantoic membrane (HET-CAM) assay (*n* = 3).

Samples	IS Score	Irritation Level
*EA* (0.5% (*w*/*v*) solution in jojoba oil)	0.00	No irritation
Vehicle (jojoba oil)	0.00	No irritation
Negative control (0.9% (*w*/*v*) NaCl)	0.00	No irritation
Positive control (1% (*w*/*v*) SLS)	6.72 ± 0.03	Moderate irritation
